# A case report of pregnancy in untreated alkaptonuria – Focus on urinary tissue remodelling markers

**DOI:** 10.1016/j.ymgmr.2021.100766

**Published:** 2021-04-29

**Authors:** L.R. Ranganath, A.M. Milan, A.C. Bay-Jensen, C.S. Thudium

**Affiliations:** a Departments of Clinical Biochemistry and Metabolic Medicine, Herlev, Denmark; b Nordic Bioscience, Herlev, Denmark

**Keywords:** Pregnancy, Alkaptonuria, Homogentisic acid, Ochronosis, Tissue biomarkers

## Abstract

A 34-year old woman with alkaptonuria had an elective pregnancy, during which she collected urine samples over the duration of her pregnancy until parturition. She had been attending the National Alkaptonuria Centre from the age of 31 years and continued to attend after delivery for a further three annual visits. Data from her NAC visits as well as urine samples collected during pregnancy were analysed. Urine CTX-1/urine creatinine, urine αCTX-I/ urine creatinine, urine CTX-II/ urine creatinine, and urine C3M/urine creatinine all showed a rapid increase early in pregnancy, returning to baseline before increasing in late pregnancy, indicating significant remodelling of bone, subchondral bone, cartilage and other organs and connective tissue rich in collagens I, II and III. The pattern of tissue remodelling in AKU pregnancy has been described for the very first time. Further research is needed to understand pregnancy in AKU.

## 1 Introduction

Alkaptonuria (AKU) (OMIM#203500) is an inherited condition present from birth, affecting men and women equally [1]. Unlike many inborn errors of metabolism where lifespan is compromised leading to early death when untreated such as in a related disorder called hereditary tyrosinaemia type 1 (HT-1), it is relatively unaffected in AKU; this allows survival into adulthood and raises issues around pregnancy and reproduction. A multitude of publications address the many manifestations of AKU, but there are no published reports of pregnancy in untreated AKU. This issue is also important since the European Medicines Agency recently authorised the use of nitisinone, a disease-modifying drug, in adults with AKU [2]. Before it can be considered for use in pregnancy, it would be necessary to understand pregnancy in AKU.

Increased appearance of homogentisic acid (HGA) due to insufficient homogentisate 1,2 dioxygenase activity follows in AKU (EC:1.13.11.5) [3]. Ochronosis, the deposition of yellow-black pigment in joint and spine cartilage, tendons, and ligaments, occurs due to unmetabolized HGA undergoing oxidation via benzoquinone acetate intermediary [4,5]. The ochronotic tissue becomes stiff and brittle and is a potential challenge to successful pregnancy, in which changes occur to create flexible and malleable tissues to enable successful pregnancy and parturition; it would therefore be important to determine if pattern of remodelling of connective tissues in the AKU pregnancy [6]. Further, protein intake in AKU patients can be altered resulting in protein malnutrition and sarcopenia, a potential further barrier to successful healthy pregnancy [7].

AKU is a severe, progressive, multisystem disease with a delayed onset of symptomatic disease, affecting the eyes, ears, laryngo-tracheal-bronchial tree, articular and fibrocartilage of joints and spine, urinary system, as well as the heart and vasculature [8,9]. The various clinical features in AKU include kidney and prostate stones, aortic stenosis, bone fractures, tendon/ligament/muscle ruptures, kyphosis, scoliosis, spinal surgery, joint replacements, besides others, and has been incorporated into a single weighted validated composite score called the Alkaptonuria Severity Score Index (AKUSSI) [10,11]. Scoring of ochronosis, the HGA-driven disease process, is an essential component of AKUSSI, and like the AKUSSI, can be used to follow changes in in individuals over time both in the untreated state and following therapy.

There are unanswered questions in pregnancy of AKU, regarding remodelling of tissues given the presence of ochronosis, and about how to counsel women who are planning pregnancy. We had the opportunity to study a single planned pregnancy in a patient attending the National Alkaptonuria Centre. As we had no idea whether AKU pregnancy was different from non-AKU pregnancy, it was decided to only collect urine samples during the pregnancy but not blood, due to ethical considerations of minimising venesections during pregnancy. Data were available from before and following the pregnancy allowing characterisation of changes in the patient around and during pregnancy. Patient was trained in how to collect, label and store random urine samples frozen, during her visit to the National Alkaptonuria Centre (NAC), and the samples were transferred to the NAC at the end of pregnancy.

## 2 Case report

A 31-year old woman with documented AKU was reviewed in 2014 in the NAC. Apart from urine turning dark she has not had any renal stones, fracture, or ruptures of Achilles tendon, muscle or any other ligaments or tendons. She had pain in the hips, knees and lower back intermittently, without affecting her activity. Previous medical history included recovered childhood asthma, visual disturbance during migraines, panic attacks and acid reflux. She consumed unrestricted dietary protein, 8 and 16 units of alcohol per week, and had briefly smoked between 2007 and 2010. Her menstrual periods were regular with menarche at 12 years. She was born of a non-consanguineous union, with neither parent having AKU or arthritis. Her family included a sister aged 39 years, a son of 4 years and a daughter of 3 months. She had never received any previous treatment for AKU. She was 1.68 m tall, weighing 58.6 kg with a BMI of 21 kg/m2. Blood pressure was 110/80 mmHg with a pulse rate of 80 per minute. Mild pigmentation was found in her ears. The rest of the general and systems examinations were all within normal limits. The 12-lead surface ECG demonstrated sinus rhythm with a normal axis. Her transthoracic echocardiogram demonstrated a LA of 31.9 ml. The mitral valve leaflets were slightly thickened but opened well with trace regurgitation. The aortic valve was normal. Whole body ^F18^PETCT showed mildly increased tracer uptake in the right patella and in the inferior tibiofibular regions bilaterally consistent with early arthropathy. Volumetric CT bone densitometry showed T score −0.36; Z score −0.32 at L1–3 vertebra, T-score of −1.27 at neck of femur (Z score −1.25), and a T score −1.47 for total hip (Z score −1.46), consistent with normal BMD in L1–3 vertebra and osteopaenia in hip. MRI of right knee was normal except for mild chronic quadriceps insertional tendinosis. MRI of spine showed early degenerative disc disease at L5/S1 with disc dehydration, focal postero-central annular bulging and an associated annular fissure. Ultrasound of abdomen and pelvis was normal except for a 2 mm gall bladder polyp on the posterior wall. ESR and FBC were normal except for mild neutropenia 1.6 × 10^9^/l (ref range 2.0–7.5). Biochemistry electrolyte and organ profiles were all normal; urine urea and creatinine were measured, the latter using Jaffe reaction in order to overcome the HGA-interference in the analyses [12].

During monitoring in the NAC, she showed further symptoms of GORD and anxiety in 2016, with increase in ^F18^PETCT uptake in both femoral condyles and right foot (in addition to both ankles). At the 2017 annual, she raised the issue of having a further pregnancy and its effect on her health; as very little was known about pregnancy in AKU, she consented to collect random urine at regular intervals during her pregnancy, which could be used to characterise changes in AKU metabolism, nutrition and tissue remodelling. At her 2018 visit, she had her third child delivered by Caesarian section 11 weeks previously, and both mother and daughter were doing well. During the pregnancy she experienced discomfort in knees, hips and lower back. The review in 2019 revealed deterioration in osteopenia, and she was asked to begin calcium 500 mg daily and 20,000 units cholecalciferol monthly; she reported pain in hips, knees, and the thoracic spine at this time. Her review in 2020 revealed peripheral sensory neuropathy. Her ^F18^PETCT scan had been stable since 2017. Assessments carried out at her annual NAC visits allowed a validated semiquantitative composite score to be derived for ochronosis and overall AKU disease, termed ochronosis and AKUSSI scores respectively. At each NAC visit she also completed Bath indices questionnaires to assess the axial spondylosis in AKU, and clinical gait analysis. A dietician discussed and assessed dietetic and nutritional aspects of her condition. At annual NAC visits circulating c-terminal telopeptide-1 (CTX-1) and procollagen-1-N-terminal telopeptide (P1NP) were measured as indicators of bone resorption and formation; serum samples were unavailable during pregnancy.

Eight random urine samples were collected by the patient during her pregnancy in 2017, at 8, 12, 16, 20, 24, 28, 32 and 36 weeks, once her pregnant state was confirmed. Metabolic analyses such as HGA, tyrosine (TYR) and phenylalanine were carried out in house both during her annual NAC visits and the random urine samples during pregnancy, as previously described [13,14]. Urine connective tissue markers were analysed and included, c-terminal telopeptide-1 (uCTX-I; marker of bone resorption), αc-terminal telopeptide-1 (uαCTX-I; marker of subchondral and young bone remodelling), c-terminal telopeptide-II (uCTX-II; a cartilage marker), and C3M (a metabolite of collagen III produced by action of matrix metalloproteinases). Urine urea and creatinine were measured on all samples, the latter using Jaffe reaction.

### 2.1 Annual NAC visit clinical data before and after pregnancy (Table 1)

**Fig. 1:**
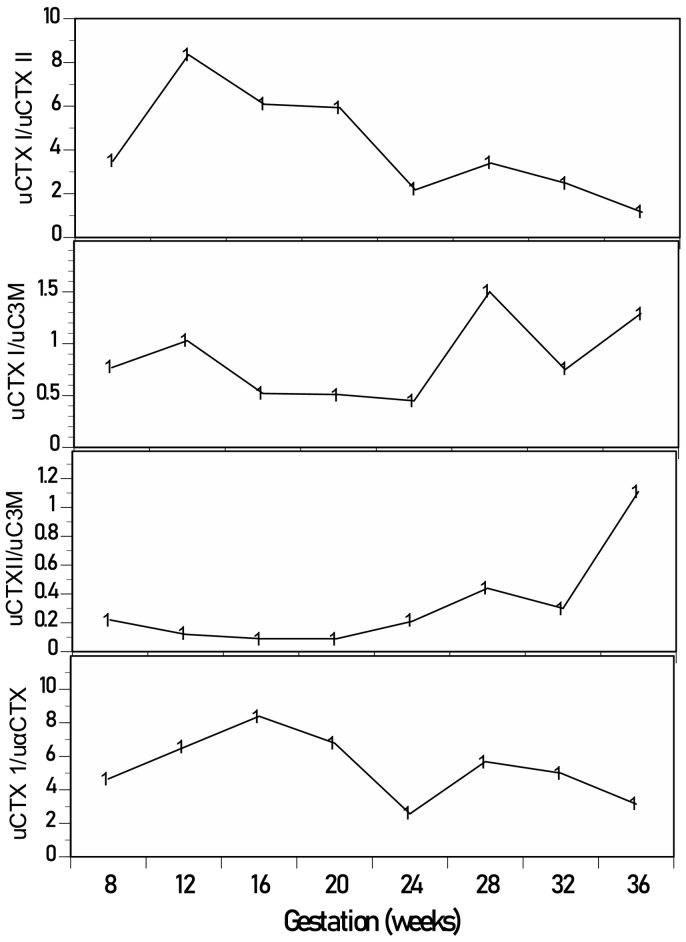
Urine tissue biomarkers inter-relationships in the patient during pregnancy in AKU showing: (A) biphasic uCTX I/uCR ratios much higher in early pregnancy; (B) biphasic uCTX II/uCR ratio showing higher later peak; (C) biphasic uαCTX/uCR ratios much higher in early pregnancy; and (D) biphasic uC3M/uCR showing a larger first peak and a much smaller late peak.

The body weight and BMI showed the expected minor variations over time and were largely stable. The data elicited from the Bath indices and KOOS questionnaires were mostly unchanged during the annual visits. Likewise, the number of steps (cadence), stride length and speed during formal gait analysis were stable throughout. There was an increase in ochronosis and AKUSSI scores over the six years of follow-up, especially starting around the time of the pregnancy. (See Fig. 1.)

**Table 1:** Clinical data showing nutritional, disease progression, Bath indices of axial spondylosis, Knee injury and Osteoarhtritis Outcomes score, and clinical gait analysis before and after pregnancy.

Year	2014	2015	2016	2017	2018	2019	2020
Weight Kg	58.6	59.8	56.7	55.5	62.1	56.1	58.4
BMI kg/M^2^	21	21.2	20.3	62.1	22	19.9	20.8
AKUSSI scores^d^	14	15	18	24	25	24	23
Ochronosis scores^d^	0	1	2	4	3	3	3
BASG^c^		0	0	0		0	0
BASDAI^c^		1.9	0.4	0.55		0.9	1.1
BASFI^c^		0.4	0	0		0.3	0
BASMI^c^		1.4	2.4	1		0	1.6
Pain^a^		92	92	89		86	72.2
Symptoms^a^	100	96	89		96	89.2	
ADL^a^		97	100	97		91.1	82.3
Sports/recreation^a^	90	100	90		100	94.1	
QoL^a^		83	81	75		88	75
Cadence^b^	123	124	127		125	126	
Stride length (m)^b^	1.26	1.29	1.38		1.38	1.41	
Speed (m/s)^b^	1.28	1.33	1.41		1.43	1.47	

a Refer to Knee Injury and Osteoarthritis Outcome Score (KOOS) questionnaire data.

b Refer to data obtained from Clinical Gait Analysis.

c Refer to data from Bath indices questionnaires.

d Refer to assessments described in REFS (10, 11, 20).

### 2.2 Pre-pregnancy biochemical and tissue turnover data from serum and urine at the annual NAC (Table 2)

The serum CTX-I and P1NP during annual visits remained stable throughout, suggesting stable coupled bone turnover. Urea and creatinine in random urine show the expected variations due to visit-to-visit differences in osmolality due to hydration. Increasing serum urea, as well as increasing urea/creatinine ratios suggest progressively increasing protein intake, especially starting around 2017. The increasing sHGA since 2018, is consistent with increased protein consumption. Similarly, urine HGA (uHGA)/urine creatinine (uCR), and urine tyrosine (uTYR)/uCR increased from 2017/2018, also supporting increased protein consumption.

**Table 2:** Serological and urinary biomarkers of biochemical and tissue turnover data before and after pregnancy.

Year	Non-AKU controls	2014	2015	2016	2017	2018	2019	2020
sCTX-1 μg/L	0.1–0.5		0.49	NA	0.44	0.47	0.37	0.36
sPINP μg/L	26–110		66.00	44.00	45	73	43	55
sUrea mM	2.5–7.8		4.8	3.4	4.3	6	6	6.4
sCreat μmol/L	50–130	69	66	59	64	59	65	72
sHGA umol/L	<3.1	8.1	13.7	12.9	10.1	15.5	14	26.9
sTYR umol/L	26–96	37	68	44	29	40	46	77
sPHE umol/L	30–76	50	NA	44	50	58	60	71
uUREA mM			190	89	70	135	259	169
uCREAT mM		9.1	7.3	3.5	1.9	3.4	6.4	4.4
uUREA/uCR			26	25.4	36.8	39.7	40.5	38.4
uHGA μmol/L	<3.1	9704	9051	4367	2815	8763	13,185	10,057
uHGA/uCR	UA	1066	1240	1248	1482	2577	2060	2286
uTYR umol/L	14–147	38.7	26.9	21	17	31	48	51
uTYR/uCR		4.25	3.68	6.0	8.95	9.12	7.50	11.59
uPHE μmol/L	10–73	43.2	NA	16	10	15	28	26
uPHE/uCR	3.4–5.0	4.75		4.57	5.26	4.41	4.38	5.91

### 2.3 Tissue biomarkers data during pregnancy (Table 3)

All urine tissue degradation markers showed a biphasic response, an early and a late response consistent with significant remodelling of bone (uCTX-1), subchondral bone (uαCTX-1), cartilage (uCTX-II) and other tissues including heart and uterus (C3M). Early peak was higher than the late peak for uCTX-1, uαCTX-1 and uC3M, whereas uCTX-II late peak was higher. Relative to each other, urine bone markers change more than cartilage early in pregnancy. Similarly, relative changes in uC3M, which includes interstitial membranes (including synovial membrane), heart and uterus are earlier than bone and cartilage. It appears that generalised bone changes and subchondral changes parallel each other during pregnancy in urine. All these tissue markers returned to pre-peak values at delivery, unlike the metabolic/nutritional ratios which remained increased at delivery.

**Table 3:** Urinary tissue turnover data during pregnancy.

Gestation (weeks)	Non-AKU controls^a^	8	12	16	20	24	28	32	36
Urine bone, cartilage and tissue turnover markers expressed per unit of urine creatinine									
uCTX-II/uCR μg/mmol	0.78 (0.70)	0.51	1.92	0.39	0.30	0.40	2.05	1.18	0.53
uC3M/uCR μg/mmol		2.32	15.63	4.52	3.40	1.95	4.65	3.89	0.48
uαCTX/uCR μg/mmol		0.39	2.46	0.28	0.26	0.34	1.23	0.58	0.20
uCTX-I/uCR μg/mmol	2.34 (1.51)	1.79	16.05	2.35	1.75	0.88	6.96	2.92	0.61

Metabolic data during pregnancy									
uUREA/uCR mmol/mmol		43.3	42.4	32.2	42.2	33.7	43.4	38.3	44.4
uHGA/uCR μmol/mmol		1009	2029	2746	1646	2839	1200	2457	1958
uTYR/uCR μmol/mmol		6.3	15.7	19.4	14.3	16.9	16.0	19.0	16.1
uPHE/uCR μmol/mmol		5.4	10.6	12.4	10.2	9.8	8.7	9.9	8.3

a Non-pregnant ranges.

## 3 Discussion

Due to the planned nature of the current pregnancy, it was possible to collect urine serially, at 8, 12, 16, 20, 24, 28, 32 and 36 weeks, once pregnancy was confirmed. The tissue remodelling marker assays in urine were not used to characterise the patient before and after pregnancy. Patient consented to collect and label these at home and store in freezer, before these samples were couriered to the National Alkaptonuria Centre in the Royal Liverpool University Hospital for analysis.

One of the questions requiring an answer is whether pregnancy affects the health of women with AKU. While the indices such as KOOS and Bath questionnaires as well as the clinical gait analysis data before and after pregnancy is more or less unaltered, it needs to be borne in mind that this is a very slowly progressive disease, which shows differing phases of progression, with an accelerated phase around the age of 50 years [10]. However, there was some increase in ochronosis and AKUSSI scores, around and after pregnancy, suggesting a pregnancy effect on the disease progression; a further study examining pregnancy in more women is needed to confirm this finding, which will help counsel women better.

Pregnancy is a physiological state in which the mother undergoes marked changes in tissues to ensure a successful outcome for the developing foetus and the mother [[15], [16], [17]]. These changes include extensive remodelling of bone, cartilage, heart, uterus and many other tissues and organs [[18], [19], [20], [21]]. This remodelling in pregnancy can be followed by examining biomarkers in urine such as those measured here, namely those of the three major collagens, I, II and III. In this AKU pregnancy, such changes were detected for all three major forms of collagen. A previous study in non-AKU pregnancy reported an increase in circulating CTX-1 and uCTX-1/uCR ratio; in the previous study, the urine ratio showed little change early in pregnancy and increased by around 50% of early levels between weeks 32–36 [22,23]. However, in the present case, the maximal changes were in early pregnancy, around 9-, 6-, 4-, and 7-times higher at 12-weeks than at 8 weeks for uCTX-I, uαCTX-I, uCTX-II and uC3M ratios relative to uCR, for reasons that are unclear. In this AKU pregnancy, subclinical AKU disease is likely to have already taken hold, as can be deduced from the MRI, PETCT, ochronosis and AKUSSI data; the hallmark of the HGA-deposition in tissues is to make these tissues more rigid and brittle, contrary to the needs during pregnancy, and we speculate whether this was a reason for the early dominant remodelling peaks, to remove rigid connective tissue to prepare for the pregnancy. Availability of serum markers would have likely clarified the situation further.

The effect of pregnancy on degenerative arthritis remains controversial with one study in rabbits [24] claiming changes while another in rat found no changes [25]. Markers similar to those used in this AKU patient have been used in studies of osteoarthritis previously [26,27]. Another short study in alkaptonuria examined tissue turnover markers and showed increases in uCTX-I/uCR and decreases in uCTX-II/uCR [28]. There are publications describing osteoporosis in AKU as well as the effects of HGA on bone resorption, but not in pregnancy [[29], [30], [31]]. A further study described AKU arthropathy as a low-turnover state [32].

This first case report in pregnancy in untreated AKU has raised more questions than answers. One previous report of very low dose nitisinone in pregnancy of AKU has been published, but did not describe tissue marker changes in AKU pregnancy itself [33,34]; however, that report of use of nitisinone in AKU pregnancy is reassuring as it found no untoward effects either in the mother or the infant, although more data is needed. Recent clinical studies of nitisinone in adult AKU excluded patients who were pregnant and terminated their participation if they became pregnant or wanted to become pregnant [35]. Since nitisinone has been approved for the treatment of adult AKU by the European Medicines Agency, there is a question about the use of nitisinone in the pregnancy of AKU, as more patients begin to be treated with nitisinone [2]. Further studies are needed to confirm if pregnancy is safe or detrimental to the untreated AKU mother, as with such data it would be possible to debate the use of nitisinone in pregnancy. Nitisinone has been used in HT-1, where it has not resulted in adverse outcomes for the mother or the foetus, although HT-1 is a fatal disease if untreated unlike AKU [36]. Further research is needed to address these questions in order to improve the management of pregnancy in AKU, and especially the use of nitisinone, and help to develop an evidence-base for effective counselling.

## Contributors

All authors contributed to analysis of the data, edited the manuscript and approved the final version.

## Funding

This work was supported by funding granted in April 2012 by the NHS England Highly Specialized Services in establishing the UK National Alkaptonuria Centre in the Royal Liverpool University Hospital. The funding source was not involved in the study design, collection, analysis and interpretation of data, the writing of the manuscript, or in the decision to submit the manuscript for publication. The authors confirm independence from the funders; the content of the article has not been influenced by the funders.

## Ethics approval

The data collected from the NAC was approved by the Institutional Audit Committee (Audit No:ACO3836).

## Data sharing statement

The authors agree to honour any reasonable request by other researchers for materials, methods or data necessary to verify the conclusion of the article.

## Declaration of Competing Interest

None.
